# Nerve conduction velocity is independently associated with bone mineral density in type 2 diabetes mellitus

**DOI:** 10.3389/fendo.2023.1109322

**Published:** 2023-02-20

**Authors:** Xiao-jing Chen, Xiao-feng Wang, Zheng-can Pan, Deng Zhang, Ke-cheng Zhu, Tao Jiang, Xiao-ke Kong, Rui Xie, Li-hao Sun, Bei Tao, Jian-min Liu, Hong-yan Zhao

**Affiliations:** ^1^ Department of Endocrine and Metabolic Diseases, Shanghai Institute of Endocrine and Metabolic Diseases, Ruijin Hospital, Shanghai Jiao Tong University School of Medicine, Shanghai, China; ^2^ Shanghai National Clinical Research Center for metabolic Diseases, Key Laboratory for Endocrine and Metabolic Diseases of the National Health Commission of the People's Republic of China, Shanghai National Center for Translational Medicine, Ruijin Hospital, Shanghai Jiao Tong University School of Medicine, Shanghai, China; ^3^ Department of Emergency, Ruijin Hospital, Shanghai Jiao Tong University School of Medicine, Shanghai, China

**Keywords:** bone mineral density, nerve conduction velocity, type 2 diabetes mellitus, diabetic peripheral neuropathy, osteoporosis

## Abstract

**Aim:**

This study investigated the association between nerve conduction velocity (NCV) and bone mineral density (BMD) in patients with type 2 diabetes mellitus (T2DM).

**Methods:**

This study retrospectively collected medical data of T2DM patients who underwent dual-energy X-ray absorptiometry and nerve conduction study at the Shanghai Ruijin Hospital, Shanghai, China. The primary outcome was the total hip BMD T-score. The main independent variables were motor nerve conduction velocities (MCVs), sensory nerve conduction velocities (SCVs), and composite Z-scores of MCV and SCV. T2DM patients were divided into total hip BMD T-scores < -1 and total hip BMD T-scores ≥ -1 groups. The association between the primary outcome and main independent variables was evaluated by Pearson bivariate correlation and multivariate linear regression.

**Results:**

195 female and 415 male patients with T2DM were identified. In male patients with T2DM, bilateral ulnar, median, and tibial MCVs and bilateral sural SCVs were lower in the total hip BMD T-score < -1 group than T-score ≥ -1 group (P < 0.05). Bilateral ulnar, median, and tibial MCVs, and bilateral sural SCVs showed positive correlations with total hip BMD T-score in male patients with T2DM (P < 0.05). Bilateral ulnar and tibial MCVs, bilateral sural SCVs, and composite MCV SCV and MSCV Z-scores were independently and positively associated with total hip BMD T-score in male patients with T2DM, respectively (P < 0.05). NCV did not show significant correlation with the total hip BMD T-score in female patients with T2DM.

**Conclusion:**

NCV showed positive association with total hip BMD in male patients with T2DM. A decline in NCV indicates an elevated risk of low BMD (osteopenia/osteoporosis) in male patients with T2DM.

## 1 Introduction

Diabetes mellitus (DM) has emerged as a major global epidemic in the 21st century. The prevalence of type 2 diabetes mellitus (T2DM) is constantly increasing worldwide and is a major healthcare concern. According to epidemiological statistics, 462 million individuals were affected with T2DM worldwide in 2017, accounting for 6.28% of the global population (1). Diabetes peripheral neuropathy (DPN) is a common complication of T2DM with prevalence rates of 35.34% in China (2), 39.3% in India (3), 42.2% in Germany (4), and 19.4% in America (5). DPN is the primary cause of foot ulceration and neuropathic pain in diabetic patients, thereby negatively impacting daily activities and adversely affecting the quality of life (6). Osteoporotic fracture is another major complication of T2D. The risk of osteoporotic fracture is significantly increased with decreased bone mineral density (BMD) (7), diabetes duration, age, previous history of fracture, and insulin use (8, 9). T2DM patients with vertebral fractures show significantly higher risk of mortality than those with T2DM alone (10).

Neurological diseases adversely affect the quality of life and alter bone metabolism (11). *However, the association between DPN and bone disease in T2DM patients remains controversial. A retrospective analysis of a cohort comprising old male veterans found that* DPN *mediated 21.1% of the diabetes-associated increased fracture risk* (12)*. Retrospective case-control studies demonstrated that DPN was an independent risk factor for osteoporosis and was independently associated with the risk of fracture and non-traumatic fractures in T2DM patients* (13, 14)*. However, there were findings that* foot bone density or fracture risk was similar in diabetic patients with and without diabetic neuropathies (15, 16). Another observational study reported bone material properties were poor in type 2 diabetes patients with DPN than those without DPN, but the association between DPN and bone health parameters were not statistically significant (17).

Two key limitations contribute to the inconsistent findings of studies on DPN: underdiagnosis of DPN in clinical settings and lack of comprehensiveness and consistency for neuropathy screening (18). Nerve conduction study (NCS) is a primary clinical screening method for DPN. Owing to its noninvasiveness, repeatability, high sensitivity, and high specificity, this method offers advantages for the assessment of neuropathic disorders (19). NCS parameters provide an objective and direct evaluation of DPN and can accurately predict disease severity (20). Myelin and axonal damages are two major lesions in DPN. In the NCS, nerve conduction velocity (NCV) mainly reflects the myelin sheath function, and action potential amplitude mainly indicates the axonal defect of nerve fibers and axon change (21). The mechanistic study revealed that myelin loss started at the prediabetic stage and preceding nerve fibers impairment (22). These results suggested that NCV is an early indicator of DPN and may be useful as an objective parameter in research studies.

It is well established that DPN and osteoporosis are two major complications of T2DM, but further investigation is warranted to determine their association. The aims of the present study were to investigate the association between NCV and BMD in T2DM patients and aimed to provide more clinical evidence concerning DPN and osteoporosis in T2DM.

## 2 Methods

### 2.1 Study design and setting

This was a retrospective study that reviewed in-patient electronic medical records between January 2017 and December 2019 at the Department of Endocrine and Metabolic Diseases, Shanghai Ruijin Hospital, a tertiary medical hospital affiliated to Shanghai Jiao Tong University School of Medicine, Shanghai, China. This study was approved by the Ethics committee of Shanghai Ruijin Hospital.

### 2.2 Study subjects

The subjects in this study were patients with T2DM that underwent dual-energy X-ray absorptiometry (DXA) and NCS. The diagnosis of T2DM was according to the World Health Organization 1999 criteria (23). Differential diagnosis of DPN included assessing the etiology of neuropathies that mimic clinical presentation of DPN, so the patients were excluded from this study if they had the following: alcohol abuse, vitamin B_12_ deficiency, neoplasia, HIV treatment, chemotherapy, amyloidosis, and genetic neuropathies (24).

### 2.3 Data collection

Demographic information, clinical and laboratory data, and antidiabetic treatments on admission or during hospitalization were retrieved from the electronic medical records. The laboratory tests were done following standard procedures in the hospital. The laboratory variables included HbA1c concentration (%), bilirubin (μmol/L), albumin (g/L), creatinine (μmol/L), triglycerides (mmol/L), total cholesterol (mmol/L), high-density lipoprotein cholesterol (mmol/L), low-density lipoprotein cholesterol (mmol/L), apolipoprotein A1 (g/L), apolipoprotein B (g/L), apolipoprotein E (mg/dL), lipoprotein (a) (g/L), and 25(OH)D (nmol/L).

### 2.4 BMD and primary outcome

BMD was measured by DXA (Lunar Expert-1313; Lunar Corp, Madison, WI, USA), and BMD T-score was calculated as the difference between the BMD of an individual and that of the reference population divided by the standard deviation (SD) of the reference population. This study evaluated the total hip BMD T-score as the primary outcome because the risk of hip fracture increased significantly in T2DM (25–27), and T2DM patients with hip fracture suffered high post-hip fracture mortality (28). According to the WHO diagnostic criteria, osteoporosis is defined as a BMD T-score lower than or equal to -2.5 SD, low bone mass (osteopenia) as a BMD T-score between -2.5 and -1 SD, and normal BMD as T-score equal to or greater than -1 SD (29). Therefore, the subjects were divided into two groups: the normal BMD group with total hip BMD T-score ≥-1 and the low BMD group, including low bone mass (osteopenia) and osteoporosis, with total hip BMD T-Score < -1.

### 2.5 The NCV and main independent variables

The NCV data, including motor nerve conduction velocities (MCVs) and sensory nerve conduction velocities (SCVs), were collected from the results of NCS by an electromyography machine (Dantec Keypoint 9033, USA) according to standard procedure. This study used the NCS values of healthy Chinese adults (18-30 years) as normal reference values: 58.8 ± 3.3 m/s for ulnar MCV, 57.8 ± 2.58 m/s for median MCV, 49.8 ± 4.39 m/s for tibial MCV, 47.44 ± 4.65 m/s for median SCV and 46.71 ± 4.17 m/s for sural SCV (30). A Z-score was developed from every NCV value based on the following formula: Z-score = (value of the individual – mean value of the normal reference)/standard deviation of the normal reference. A composite MCV Z-score was calculated as the following formula: [(bilateral ulnar MCV Z-scores) + (bilateral median MCV Z-scores) + (bilateral tibial MCV Z-scores)]/6, a composite SCV Z-score calculated as: [(bilateral median SCV Z-scores) + (bilateral sural MCV Z-scores)]/4, and a composite MSCV Z-score calculated as the composite MCV Z-score + SCV Z-score (31, 32). The main independent variables in this study were MCVs of the bilateral ulnar, median, and tibial nerves, SCVs of bilateral median and sural nerves, and composite Z-scores.

### 2.6 Statistical analysis

Categorical variables were displayed as numbers and percentages. Continuous variables were checked for normal distribution by the Kolmogorov–Smirnov test and were summarized as the arithmetic mean ± SD if with normal distribution and as the median and interquartile range (IQR) if with no normal distribution. The characteristics between T-score < -1 and T-score ≥ -1 groups were compared by chi-square test for categorical variables and by two-way ANOVA and t-test for continuous variables as appropriate.

This study hypothesized that NCV is associated with BMD in T2DM and applied a multi-step modeling process to identify the association between NCV and total hip BMD. Firstly, Pearson correlations were conducted to evaluate the bivariate relationship between total hip BMD T-score and each MCV, SCV, and composite Z-score. Subsequently, univariate linear regression was performed to assess the univariate association between total hip BMD T-score and age, BMI, laboratory variables, and each MCV, SCV, and composite Z-score, respectively. Finally, stepwise multivariate linear regression was performed to identify the independent association between NCV and total hip BMD T-score and adjust the effects of covariates. MCVs, SCVs, and composite Z-scores were independent variables and separately entered into multivariate linear regression models to avoid collinearity. Covariates in multivariate linear regression included smoke, alcohol, T2DM treatments, HbA1c, and variables that were significantly associated with total hip BMD T-score in univariate linear regression or possibly associated with BMD from scientific/clinical knowledge. The sample size of our study was large enough and already met the ten events per variable principle, which is a widely advocated minimal criterion of the sample size to avoid overfitting in regression analysis (33). No collinearity was observed among covariates (VIF < 3.0 and r < 0.7). Statistical analysis was performed with IBM SPSS Statistics 27.

## 3 Results

### 3.1 The clinical characteristics compared according to total hip BMD T-score separately for males and females with T2DM

This study identified 195 female and 415 male patients with T2DM. The prevalence of T-score < -1 was higher in females than males among T2DM patients (29.23% *vs*. 12.05%, P < 0.001). The clinical characteristics were compared between total hip BMD T-score < -1 and T-score ≥ -1 groups separately for male and female patients (**Table 1**). The age was older in the T-score < -1 group than in the T-score ≥ -1 group in female patients with T2DM (P < 0.001). The BMI was lower in T-score < -1 group than in the T-score ≥ -1 group in the female and male patients with T2DM (P < 0.001). The HbA1c level was lower in T-score < -1 group than in the T-score ≥ -1 group in the female patients with T2DM (P < 0.05). In male patients with T2DM, compared to the T-score ≥ -1 group, the T-score < -1 group had a lower level of left ulnar MCV, right ulnar MCV, left median MCV, right median MCV, left tibial MCV, right tibial MCV, left sural SCV, right sural SCV, composite MCV Z-score, composite SCV Z-score, and composite MSCV Z-score, respectively (P < 0.05). However, the two groups did not show significant differences in the MCVs and SCVs in female patients with T2DM.

**Table 1:** Clinical characteristics compared between total hip BMD T-score < -1 and ≥ -1 groups for female and male patients with T2DM.

	Females (195)			Males (415)		
	T-score < -1 (57)	T-score ≥ -1 (138)	P	T-score < -1 (50)	T-score ≥ -1 (365)	P
Age (year)	66.39 ± 7.59	59.02 ± 10.84	< 0.001	58.26 ± 11.12	55.63 ± 10.65	0.1
Height (cm)	159.04 ± 5.89	161.11 ± 5.88	0.03	171.75 ± 6.2	172.68 ± 5.2	0.25
BMI (kg/m^2^)	22.81 ± 3.4	25.39 ± 3.96	< 0.001	23.51 ± 2.44	26.04 ± 3.28	< 0.001
Smoke (%)	0 (0)	6 (4.3)	0.11	33 (66.0)	199 (54.5)	0.13
Alcohol (%)	12 (21.1)	24 (17.4)	0.55	15 (30.0)	145 (39.7)	0.19
Antidiabetic treatment						
Sulfonylureas n (%)	25 (43.9)	51 (37.0)	0.37	18 (36.0)	129 (35.3)	0.93
Glinides n (%)	8 (14.0)	15 (10.9)	0.53	6 (12.0)	53 (14.5)	0.63
Biguanides n (%)	32 (56.1)	101 (73.2)	0.02	27 (54.0)	251 (68.8)	< 0.05
Acarbose n (%)	33 (57.9)	66 (47.8)	0.20	29 (58.0)	158 (43.3)	0.05
TZD n (%)	5 (8.8)	15 (10.9)	0.66	2 (4.0)	25 (6.8)	0.44
Insulin n (%)	35 (61.4)	75 (54.3)	0.37	35 (70.0)	186 (51.0)	< 0.05
HbA1c (%)	7.9 ± 1.26	8.41 ± 1.98	0.03	8.18 ± 1.61	8.22 ± 1.83	0.89
Bilirubin (μmol/L)	5.53 ± 4.83	4.95 ± 4.92	0.45	12.54 ± 6.08	12.56 ± 4.62	0.98
Albumin (g/L)	38.07 ± 3.1	38.28 ± 2.83	0.64	38.46 ± 3.99	39.48 ± 4.21	0.11
Serum creatinine (μmol/L)	58.11 ± 15.65	57.96 ± 12.32	0.94	77.71 ± 23.52	78.17 ± 25.41	0.9
Triglycerides (mmol/L)	1.84 ± 1.46	2.16 ± 2.18	0.32	2.02 ± 1.24	2.17 ± 1.9	0.57
Total cholesterol (mmol/L)	4.61 ± 1.11	4.68 ± 1.06	0.71	4.53 ± 1.15	4.32 ± 1.13	0.22
HDL (mmol/L)	1.24 ± 0.28	1.22 ± 0.32	0.58	1.05 ± 0.27	1.03 ± 0.22	0.55
LDL (mmol/L)	2.75 ± 0.95	2.78 ± 0.85	0.83	2.75 ± 0.89	2.58 ± 0.86	0.19
Apolipoprotein A1 (g/L)	1.33 ± 0.19	1.33 ± 0.22	0.93	1.2 ± 0.16	1.19 ± 0.2	0.75
Apolipoprotein B (g/L)	0.87 ± 0.26	0.88 ± 0.22	0.76	0.87 ± 0.21	0.84 ± 0.23	0.43
Apolipoprotein E (mg/dL)	3.97 ± 1	4.22 ± 1.46	0.23	3.81 ± 1.16	3.95 ± 1.64	0.56
Lipoprotein (a) (g/L)	0.24 ± 0.23	0.22 ± 0.21	0.57	0.2 ± 0.2	0.18 ± 0.22	0.55
25(OH)D (nmol/L)	42.69 ± 16.46	43.85 ± 16.53	0.66	47.14 ± 19.95	46.82 ± 18.28	0.91
**NCV**						
Left ulnar MCV (m/s)	56.06 ± 5.4	55.65 ± 4.49	0.58	50.69 ± 6.88	52.94 ± 5.7	0.01
Right ulnar MCV (m/s)	56.06 ± 5.36	56.41 ± 4.92	0.66	51.71 ± 6.55	53.7 ± 5.39	0.04
Left median MCV (m/s)	56.46 ± 5.84	55.82 ± 5.1	0.45	53.38 ± 6.43	55.38 ± 4.9	0.04
Right median MCV (m/s)	55.18 ± 5.75	55.35 ± 5.51	0.85	52.91 ± 6.57	54.73 ± 5.19	0.03
Left tibial MCV (m/s)	44.52 ± 5.59	44.09 ± 5.55	0.62	42.27 ± 4.76	44 ± 5.39	0.03
Right tibial MCV (m/s)	43.76 ± 5.63	44.08 ± 5.32	0.71	41.49 ± 5.51	44.02 ± 5.36	0.01
Left median SCV (m/s)	52.91 ± 11.16	52.91 ± 10.16	1.00	51.58 ± 12.01	54.72 ± 9.14	0.08
Right median SCV (m/s)	52.54 ± 9.89	51.34 ± 11.39	0.49	48.84 ± 15.19	53 ± 10.67	0.07
Left sural SCV (m/s)	53 ± 10.08	50.44 ± 12.54	0.17	45.46 ± 13.34	50.3 ± 11.04	< 0.001
Right sural SCV (m/s)	50.44 ± 11.66	49.58 ± 13.22	0.67	42.68 ± 14.6	49.47 ± 11.9	< 0.001
Composite MCV Z-score	-0.96 ± 1.38	-1 ± 1.24	0.85	-1.97 ± 1.67	-1.35 ± 1.33	< 0.001
Composite SCV Z-score	1.17 ± 1.96	0.9 ± 2.22	0.43	-0.02 ± 2.77	1.07 ± 2.03	0.01
Composite MSCV Z-score	0.21 ± 3.21	-0.1 ± 3.22	0.54	-1.99 ± 4.21	-0.28 ± 3.13	0.01
Dual-energy X-ray results						
L1–4 BMD (g/m^2^)	0.93 ± 0.16	1.15 ± 0.17	< 0.001	1.04 ± 0.13	1.22 ± 0.16	< 0.001
L1–4 BMD T score	-1.5 ± 1.32	0.29 ± 1.4	< 0.001	-0.32 ± 1.06	1.17 ± 1.36	< 0.001
FN BMD (g/m^2^)	0.71 ± 0.08	0.92 ± 0.11	< 0.001	0.76 ± 0.07	0.98 ± 0.13	< 0.001
FN BMD T score	-1.82 ± 0.67	-0.1 ± 0.96	< 0.001	-1.67 ± 0.59	0.02 ± 0.97	< 0.001
WT BMD (g/m^2^)	0.52 ± 0.1	0.75 ± 0.15	< 0.001	0.58 ± 0.1	0.81 ± 0.2	< 0.001
WT BMD T score	-2.41 ± 0.64	-0.86 ± 1	< 0.001	-2.03 ± 0.68	-0.41 ± 1.16	< 0.001
Trochanteric BMD (g/m^2^)	0.59 ± 0.08	0.78 ± 0.1	< 0.001	0.66 ± 0.07	0.86 ± 0.14	< 0.001
Trochanteric T score	-1.46 ± 0.76	0.17 ± 0.86	< 0.001	-1.42 ± 0.56	0.34 ± 0.9	< 0.001
Total hip BMD (g/m^2^)	0.75 ± 0.08	0.99 ± 0.1	< 0.001	0.79 ± 0.06	1.05 ± 0.12	< 0.001
Total hip T score	-1.76 ± 0.69	0.18 ± 0.81	< 0.001	-1.54 ± 0.45	0.42 ± 0.89	< 0.001

BMD, Bone mineral density; T2DM, type 2 diabetes mellitus (T2DM); TZD, Thiazolidinedione; HDL, High-density lipoprotein; LDL, Low-density lipoprotein; NCV, Nerve conduction velocity; MCV, Motor nerve conduction velocity; SCV, Sensory nerve conduction velocity; MSCV, Motor and sensory nerve conduction velocity; L1–4, Lumbar spine 1–4; FN, Femoral neck; WT, Ward’s triangle.

### 3.2 The positive correlation between NCV and total hip BMD T-score in male patients with T2DM

**Figure 1:**
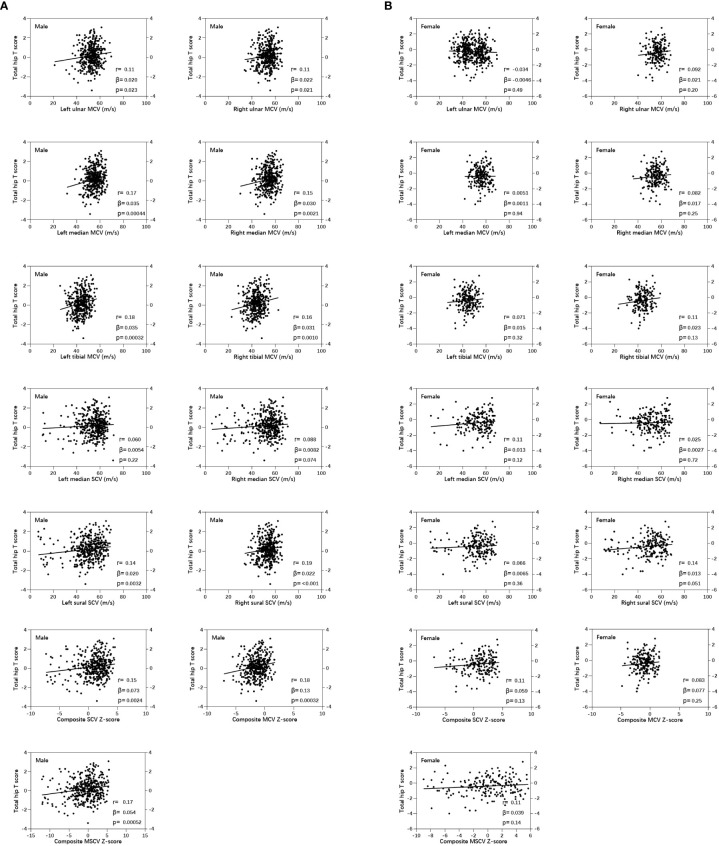
Pearson correlations showed the correlation between nerve conduction velocity and total hip bone mineral density (BMD) T-score in male **(A)** and female **(B)** patients with T2DM.

In male patients with T2DM, a significant positive bivariate correlation existed between total hip BMD T-score and variables: left ulnar MCV, right ulnar MCV, left median MCV, right median MCV, left tibial MCV, right tibial MCV, left sural SCV, right sural SCV, composite MCV Z-score, composite SCV Z-score, and composite MSCV Z-score, respectively (P < 0.05) (**Figure 1A**). While in female patients with T2DM, there was no significant bivariate correlation between total hip BMD T-score and NCV (**Figure 1B**).

### 3.3 The independent association between NCV and total hip BMD T-score in male patients with T2DM

**Figure 2:**
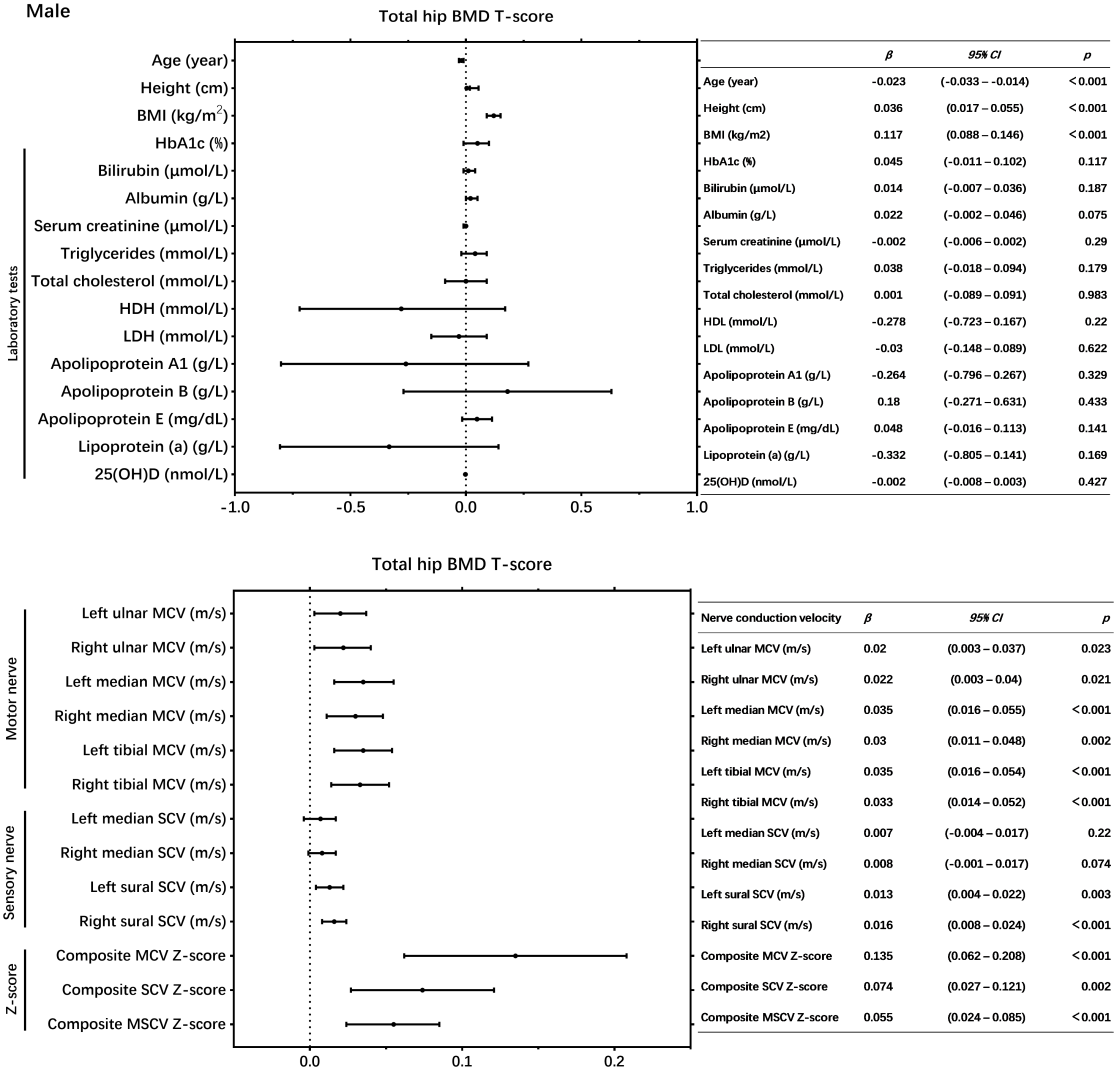
Univariate linear regression showed the association between nerve conduction velocity and total hip bone mineral density (BMD) T-score in male patients with T2DM.

**Figure 3:**
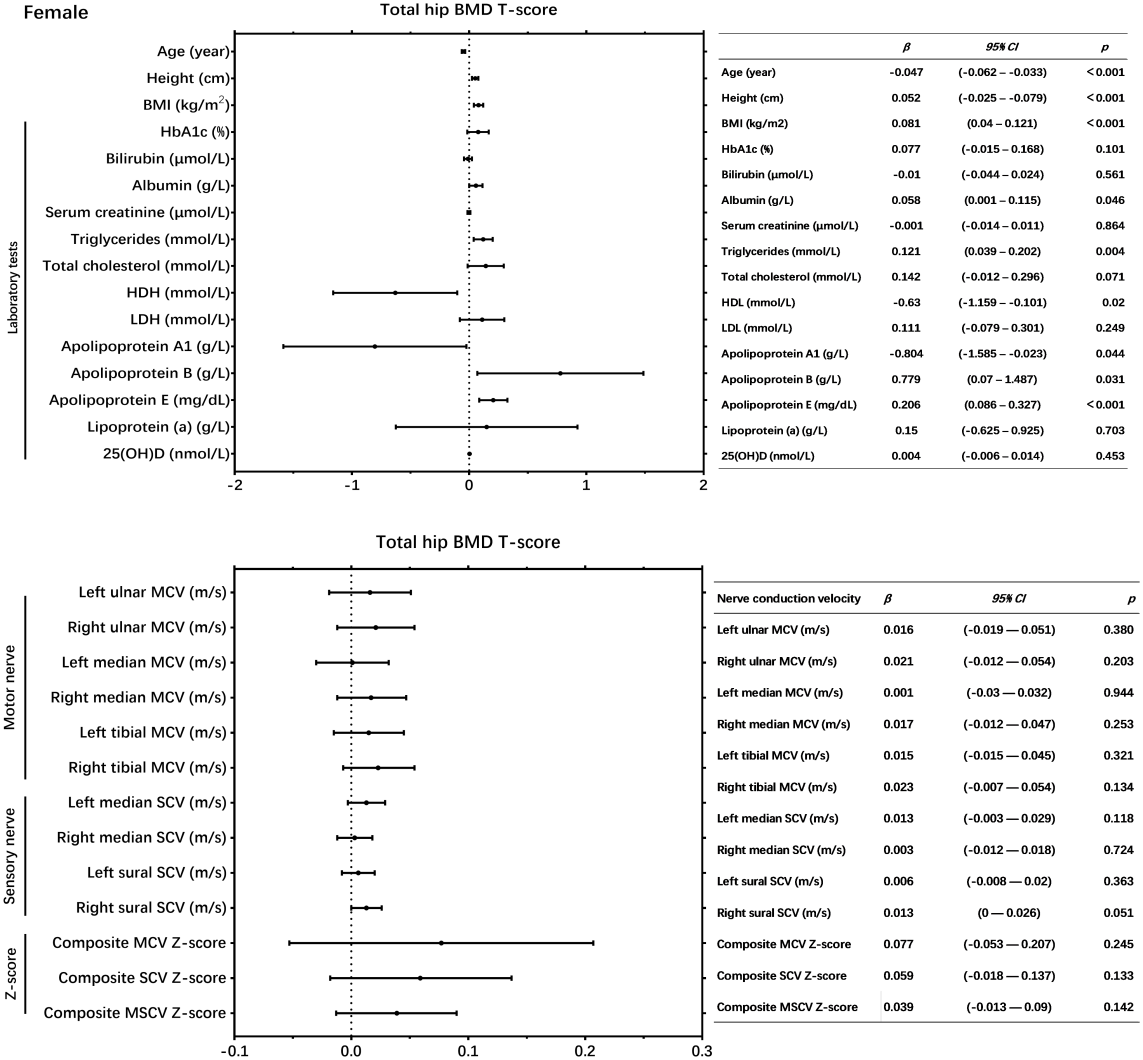
Univariate linear regression showed the association between neSrve conduction velocity and total hip bone mineral density (BMD) T-score in female patients with T2DM.

In male patients with T2DM, univariate linear regression analysis revealed that each of the following variables was significantly correlated with the total hip BMD T score: age, BMI, left ulnar MCV, right ulnar MCV, left median MCV, right median MCV, left tibial MCV, right tibial MCV, left sural SCV, right sural SCV, composite MCV Z-score, composite SCV Z-score and composite MSCV Z-score (P < 0.05) (**Figure 2**). While in female patients with T2DM, NCV was not significantly correlated with total hip BMD T score (**Figure 3**).

In male patients with T2DM, multivariable regression models, adjusting for age, BMI, and other confounding variables, revealed that each of the following NCVs was independently and positively associated with the total hip BMD T score: left median MCV, right median MCV, left tibial MCV, right tibial MCV, left sural SCV, right sural SCV, composite MCV Z-score, composite SCV Z-score, and composite MSCV Z-score (P < 0.05) (**Table 2**). Multivariable regression revealed no other covariables were significantly associated with the total hip BMD T score in male patients with T2DM.

**Table 2:** Association of nerve conduction velocity and total hip BMD T-score in male patients with T2DM by multivariable linear regression.

Nerve conduction velocity	*β*	*95% CI*	*P*	*Adjusted R2*
Motor nerve conduction velocities				
Left ulnar MCV (m/s)				
Model 1	0.012	(-0.005 – 0.028)	0.162	0.158
Model 2	0.009	(-0.008 – 0.026)	0.287	0.164
Right ulnar MCV (m/s)				
Model 1	0.011	(-0.006 – 0.029)	0.217	0.157
Model 2	0.011	(-0.007 – 0.030)	0.240	0.165
Left median MCV (m/s)				
Model 1	0.022	(0.003 – 0.041)	0.025	0.164
Model 2	0.028	(0.007 – 0.048)	0.008	0.168
Right median MCV (m/s)				
Model 1	0.02	(0.001 – 0.038)	0.035	0.163
Model 2	0.022	(0.003 – 0.040)	0.021	0.168
Left tibial MCV (m/s)				
Model 1	0.022	(0.004 – 0.04)	0.018	0.166
Model 2	0.022	(0.004 – 0.04)	0.018	0.166
Right tibial MCV (m/s)				
Model 1	0.019	(0 – 0.037)	0.044	0.162
Model 2	0.019	(0 – 0.037)	0.044	0.162
Sensory nerve conduction velocities				
Left median SCV (m/s)				
Model 1	0	(-0.01 – 0.011)	0.968	0.154
Model 2	-0.02	(-0.013 – 0.009)	0.733	0.156
Right median SCV (m/s)				
Model 1	0.004	(-0.005 – 0.013)	0.409	0.155
Model 2	0.003	(-0.006 – 0.012)	0.483	0.162
Left sural SCV (m/s)				
Model 1	0.009	(0.001 – 0.018)	0.033	0.163
Model 2	0.009	(0.001 – 0.018)	0.033	0.163
Right sural SCV (m/s)				
Model 1	0.013	(0.005 – 0.021)	0.001	0.175
Model 2	0.013	(0.005 – 0.021)	0.001	0.175
Composite Z-scores				
Composite MCV Z-score				
Model 1	0.085	(0.013 – 0.158)	0.021	0.165
Model 2	0.088	(0.016 – 0.160)	0.017	0.169
Composite SCV Z-score				
Model 1	0.050	(0.003 – 0.096)	0.036	0.163
Model 2	0.050	(0.003 – 0.096)	0.036	0.163
Composite MSCV Z-score				
Model 1	0.037	(0.006 – 0.067)	0.019	0.165
Model 2	0.037	(0.006 – 0.067)	0.019	0.165

Model 1: adjusted for age and BMI.

Model 2: additionally adjusted for smoke, alcohol, sulfonylureas, glinides, biguanides, acarbose, thiazolidinedione, insulin, HbA1c, bilirubin, albumin, serum creatinine, triglycerides, high-density lipoprotein.

BMD, Bone mineral density; T2DM, type 2 diabetes mellitus (T2DM); MCV, Motor nerve conduction velocity; SCV, Sensory nerve conduction velocity; MSCV, Motor and sensory nerve conduction velocity.

## 4 Discussion

Osteoporotic fracture is a critical survival factor for T2DM, but the risk of which is often underestimated. BMD decline increases osteoporotic fracture risk in T2DM (7). Thus, it is important to identify T2DM patients at high risk of osteoporosis and osteoporotic fractures and explore factors responsible for or associated with BMD changes. In the current study, we found that NCV is independently and positively associated with BMD in male patients with T2DM, which highlighted NCV decline as a high-risk factor for low BMD (osteopenia/osteoporosis).

The previous studies have yet to reach a consistent conclusion about the association between DPN and osteoporosis in DM. A meta-analysis included five studies investigating the association between DPN and BMD in T1DM or T2DM, 4 of which found no effect of DPN on BMD, and this study highlighted the major limitation for analysis is the different diagnostic criteria for DPN among five studies (34). *DPN is a heterogeneous clinical condition and an exclusive diagnosis. It needs multistep screening processes to exclude other peripheral neuropathies, including alcoholism, vitamin B12 deficiency, autoimmune diseases, tumors, and infection.* DPN varies considerably in the screening and diagnostic criteria, including questionnaires, electrophysiological methods, imaging diagnostic tools, and hierarchical classification schemes (35), leading to heterogeneity across studies. In addition, more than half of physicians did not adhere to standardized multistep screening and diagnosis of DPN (36). Nearly half of DPN cases may be asymptomatic (37, 38), thereby complicating the recognition by the patients (39) and their physicians (40). These facts lead to heterogeneity in the diagnostic rate of DPN in T2DM among different clinical settings and make it difficult to reach consistent and certain conclusions in DPN studies.

The NCS is a sensitive, specific, non-invasive, and quantitative screening tool for DPN, and it has been recommended as the gold diagnostic standard by expert consensus (41). The NCS result has been found to be associated with glycemic control (42), duration of diabetes (43), and cognitive dysfunction in T2DM (44). Therefore, this study used NCVs as important parameters and found their positive association with total hip BMD in male patients with T2DM. This finding supports the advantage of using NCS in DPN research.

In the present study, bilateral tibial MCV and sural SCV were independently associated with total hip BMD; however, such associations were not observed for ulnar MCV and median SCV. This finding is consistent with the phenomenon that the symptom of DPN starts at the toes, feet, and lower legs (45). In NCS, the tibial was the most sensitive nerve compared to the peroneal and sural for diagnosis of polyneuropathy (46). The composite Z-scores are a summary parameter that can quantify the overall abnormality of NCVs. It has been demonstrated to have higher specificity and sensitivity for evaluating polyneuropathies than individual nerve MCV or SCV results (47). Our study found that each of the composite MCV, SCV, and MSCV Z-scores was independently and positively associated with total hip BMD T-score in male patients with T2DM, which shows the association between overall abnormality of NCV and total hip BMD decline.

Sympathectomy in diabetic mice would induce the increased stromal cell-derived factor 1 level and decreased bone regeneration, but the specific molecular mechanism is unknown (48). Besides, no other study deeply evaluates the underlying mechanism of distal peripheral neuronal regulation on bone homeostasis and remodeling in diabetes. However, the regulation of neurotrophic and neurovascular bone metabolism suggests the contribution of neuropathy to decreased bone health in DM. Peripheral nerves promote bone remodeling and regeneration, which is mediated by osteogenesis induction of nerve growth factor and axon guidance molecule (49). The diabetes adults also have decreased levels of two sensory neuropeptides with bone anabolic activity, namely substance P and calcitonin gene-related peptide (CGRP) (50). In diabetes, peripheral neuropathy was associated with blood vessel damage (51) and abdominal obesity (52), which might mediate the interplay between DPN and bone disease. More clinical studies are needed to examine the association between DPN and bone disease in diabetes.

It is noteworthy that the association between NCV and total hip BMD was not observed in female type 2 diabetes in this study. Some studies provided clues to explain this phenomenon. Epidemiological studies showed that, in T2DM, the male was more susceptible to microvascular complications (53) and had an earlier onset of DPN than the female (54). In the diabetic model, male mice had a slower level of MCV than female mice at 16-weeks of diabetes (55). In addition, neuroactive steroids regulate the peripheral nervous system in a sex-dependent manner (56). In the male rat, short-term diabetic status affected the respiratory chain complex IV and ATP levels and decreased the axoplasm protein contents and mRNA levels of kinesin family member protein 1A, 5B, and 5A in axons of dorsal root ganglion neurons, which was mediated by decreased levels of neuroactive steroid, including testosterone, dihydrotestosterone, and allopregnanolone. Meanwhile, those changes were not observed in female rats (57). The above studies suggest the impact of gender or sex hormone on the development of DPN, and more mechanistic studies are needed to explain the gender-based difference in the association between DPN and osteoporosis in type 2 diabetes.

Undoubtedly, there were several limitations in the present study. Firstly, NCV normal values were referred from relatively young, healthy Chinese. However, a significant difference did not exist because the NCV reference was homogeneous to all the subjects. Secondly, fall is an important external factor responsible for fracture in T2DM, but fall data was unavailable for our database. Thirdly, this study did not include osteoporotic fracture and failed to evaluate bone disease in T2DM comprehensively. More large-scale cohort studies are required to yield more comprehensive clinical data and determine the cause-to-consequence effect between DPN and osteoporosis and osteoporotic fracture in type 2 diabetes.

## 5 Conclusion

In summary, this study revealed that NCV was independently and positively associated with total hip BMD in male patients with T2DM, however, this association was not observed in female patients with T2DM. Hence, NCV decline might be a high-risk indicator for low BMD (osteopenia/osteoporosis) in male patients with T2DM. Mechanistic and epidemiological studies are warranted to determine the causal effect between DPN and low BMD in T2DM.

## Data availability statement

The original contributions presented in the study are included in the article/supplementary material. Further inquiries can be directed to the corresponding authors.

## Ethics statement

The studies involving human participants were reviewed and approved by Ruijin Hospital Ethics Committee Shanghai JiaoTong University School of Medicine. Written informed consent for participation was not required for this study in accordance with the national legislation and the institutional requirements.

## Author contributions

H-yZ and J-mL were the guarantors of this work and, as such, had full access to all the data in the study and took responsibility for the integrity of the data and the accuracy of the data analysis and was involved in the conception and design of the study. X-jC collected and analyzed the data. X-fW, Z-cP, DZ, K-cZ, TJ, X-kK, RX, L-hS, and BT were involved in the collection and interpretation of data. X-jC drafted the manuscript. J-mL modified the manuscript. H-yZ revised the manuscript for important intellectual content. All authors contributed to the article and approved the submitted version.
